# Hypertensive crisis: clinical characteristics of patients with hypertensive urgency, emergency and pseudocrisis at a public emergency department

**DOI:** 10.31744/einstein_journal/2019AO4685

**Published:** 2019-08-27

**Authors:** Angela Maria Geraldo Pierin, Carime Farah Flórido, Juliano dos Santos

**Affiliations:** 1 Universidade de São Paulo, São Paulo, SP, Brazil.; 2 Escola de Enfermagem, Universidade de São Paulo, São Paulo, SP, Brazil.; 3 Instituto Nacional de Câncer, Rio de Janeiro, RJ, Brazil.

**Keywords:** Hypertension, Emergency medical services, Emergency treatment, Prevalence

## Abstract

**Objective:**

To assess patients with hypertensive crisis, classified as urgency, emergency or pseudocrisis, and identify the associated variables.

**Methods:**

We evaluated a total of 508 patients (57% women; 56.3±13.8 years old) with hypertensive crisis (diastolic blood pressure of 120mmHg), aged 18 years or over, seen at the emergency department of a public general hospital.

**Results:**

The prevalence of hypertensive crises was 6/1,000; in that, 71.7% presented hypertensive urgency, 19.1% hypertensive emergency, and 9.2% hypertensive pseudocrisis. In the multinominal logistic regression, pseudocrisis and urgency conditions were compared to hypertensive emergency. Therefore, the presence of pain (OR: 55.58; 95%CI: 10.55-292.74) except chest pain and headache, and emotional problems (OR: 17.13; 95%CI: 2.80-104.87) increased the likelihood of hypertensive pseudocrisis. Age >60 years (OR: 0,32; 95%CI: 0.10-0.96) and neurologic problems (OR: 1.5.10^-8^; 95%CI: 1.5.10^-8^-1.5.10^-8^) protected against hypertensive pseudocrisis. The comparison of hypertensive urgency with hypertensive emergency showed that age >60 years (OR: 0.50; 95%CI: 0.27-0.92), neurologic (OR: 0.09; 95%CI: 0.04-0.18) and emotional problems (OR: 0.06; 95%CI: 4.7.10^-3^-0.79) protected against hypertensive urgency. Moreover, only headache (OR: 14.28; 95%CI: 3.32-61.47) increased the likelihood of hypertensive urgency.

**Conclusion:**

Advanced age and neurological problems were associated to hypertensive emergency. Headache was associated with hypertensive urgency. Pain and emotional problems were associated with hypertensive pseudocrisis. Our results can contribute to identifying patients with hypertensive crisis who seek emergency services.

## INTRODUCTION

The World Health Organization points out that cardiovascular diseases account for approximately 17 million deaths per year, and complications from hypertension account for 9.4 million of these deaths.^(^^1^^)^ Hypertensive crisis is one of the major acute complications of hypertension, resulting in an emergency admission to hospital.^(^^2^^)^ A review article on the subject shows that the prevalence and characteristics of patients with hypertensive crisis have changed in the last four decades. However, morbidity and mortality are still significant.^(^^3^^)^

Hypertensive crisis is characterized by severe and abrupt elevation of blood pressure,^(^^4^^)^ usually defined by diastolic pressure values above 120mmHg. It is classified as a hypertensive urgency when there is no end-organ damage, and as hypertensive emergency when there is a risk of death evidenced by end-organ damage.^(^^5^^)^ Hypertensive urgency and hypertensive emergency should be distinguished from a hypertensive pseudocrisis, which is characterized by a transient elevation of the blood pressure during painful or emotional events, such as headache, rotational dizziness, anxiety, or panic syndrome.^(^^6^^)^ The treatment of a hypertensive urgency consists of a gradual reduction of blood pressure using oral medication, whereas in a hypertensive emergency, intravenous therapy is indicated for a faster reduction in blood pressure.^(^^7^^)^ As to hypertensive pseudocrisis, the treatment is focused on symptoms, and the subject is little explored in the literature on hypertensive crisis.

Brazilian literature is still poor in studies on the characteristics of patients with hypertensive crisis in emergency services and their associated factors. The work of the multiprofessional team involves cooperation for the best management and treatment, due to the complexities of the urgency and emergency situations presented by this clinical condition, and the lack of knowledge on hypertensive crisis can have an unexpected impact on the health team. Therefore, the present study is considered relevant, because it contributes to the clarification of the theme, broadening knowledge in the country.

## OBJECTIVE

To evaluate patients with hypertensive crisis, classified as hypertensive urgency, hypertensive emergency, or hypertensive pseudocrisis; and to identify the associated variables.

## METHODS

This was a cross-sectional, retrospective study, with a quantitative approach, conducted in the emergency department of a municipal hospital in the city of São Vicente, located on the south coast of the State of São Paulo. The city of São Vicente has a fixed population of approximately 350 thousand inhabitants. It is a public hospital, and its emergency unit is specialized in adult and pediatric emergency care. To identify patients with hypertensive crisis, all records of patients seen in the emergency department for adults and in the clinical specialty were evaluated in the period from January to June 2015, in a total of 83,774 medical consultations.

A total of 508 patients who met the following inclusion criteria were identified: diastolic blood pressure ≥120mmHg and age ≥18 years. Of the 508 patients identified, 435 were included, and 73 were excluded due to absence of information in the patients' records, which rendered impossible to identify the variables of the study or to classify the type of hypertensive crisis.

The independent variables studied were age, sex, blood pressure, reason for consulting an emergency service, treatment, identified medical diagnosis, and referral. The outcome variables were hypertensive emergency, hypertensive urgency, and hypertensive pseudocrisis. Classification of hypertensive crisis was performed by specialists, according to the Seventh Report of the Joint National Committee on Prevention, Detection, Evaluation, and Treatment of High Blood Pressure (JNC 7),^(^^5^^)^ with attention and critical sense based mainly on the signs and symptoms of patients. Patients with signs and symptoms, such as musculoskeletal pain, stomach pain, painful swallowing, heartburn and general pain were considered as presenting a hypertensive pseudocrisis, except for those with chest pain and unspecified headache, which were classified as a hypertensive urgency or a hypertensive emergency, depending on other signs and symptoms that evidenced or not end-organ damage, and/or the patients had medical diagnosis records that allowed the identification of emotional problems, such as stress-related spikes and emotional lability. Patients with signs and symptoms that evidenced end-organ damage, such as neurological problems, including decreased motor strength, paresthesia, confusion, mental disorientation, chest pain, and dyspnea, as well as medical diagnoses of stroke, acute myocardial infarction and acute pulmonary edema, were classified as a hypertensive emergency. Hypertensive urgencies were classified according to signs and symptoms that did not characterize hypertensive pseudocrisis or did not evidence end-organ damage. Data collection was performed using the information on the emergency department records. Thus, after the laboratory tests conducted according to the customary practice of the emergency department, the patients were reassessed by the medical team, who recorded the diagnosis that corresponded to the tests' results, allowing the classification of the hypertensive crisis as urgency or emergency in the present study.

The values entered on the patients' emergency department records were used to characterize blood pressure. The variable was expressed by mean and standard deviation in mmHg. The blood pressure measurement in the emergency department in which the study was performed is routinely performed by physicians and the nursing team, using the indirect method, with auscultatory technique and the use of aneroid devices that are periodically evaluated for calibration. To identify the clinical manifestations, clinical management and treatment of patients, all information contained in the emergency department records was considered, through judicious and careful reading, by two nurses who worked in the service. The study was approved by the Ethics Committee, CAAE: 53896016.9.0000.5392, with the agreement of the head of the emergency department.

The results were expressed as mean±standard deviation (for normal distribution variables), and as absolute numbers and percentage for categorical variables. For the parametric data, an analysis of variance (ANOVA) was used. The Pearson's coefficient was used to evaluate proportions. Values of p≤0.05 were considered statistically significant. Multivariate analysis was performed by multinomial logistic regression to identify the independent variables that remained associated with the hypertensive crisis, such as age over 60 years, pain (except headache and chest pain), headache, neurological problems and emotional problems, on the chances of developing pseudocrisis or hypertensive urgency in relation to hypertensive emergency. In this model, the statistical test used was the Pearson's χ^2^ test. The data obtained were entered into a Microsoft Excel^®^ worksheet using the Statistical Package for Social Science (SPSS), version 22, and the program R version 3.3.3.

## RESULTS

**Figure 1:**
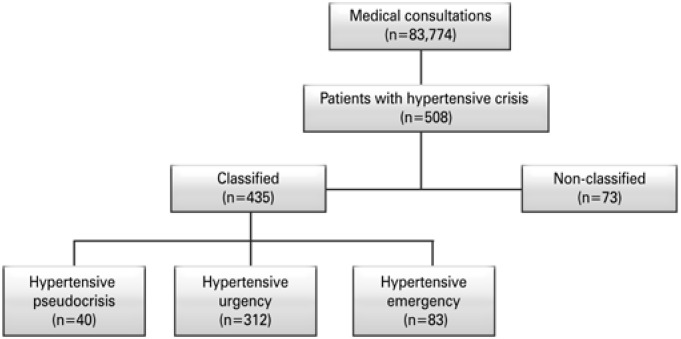
Population included in the study

Of the total number of patients retrospectively evaluated in the emergency department, during the 6-month period, the prevalence of hypertensive crisis was 6/1,000. Most were classified as hypertensive urgencies, followed by hypertensive emergencies and hypertensive pseudocrisis, as shown in figure 1 .

Data in table 1 show that most patients were female, and the mean age was in the range of the fifth decade of life. Patients with hypertensive emergency were significantly older than those with hypertensive urgency and hypertensive pseudocrisis, but there was no significant difference in relation to sex in these three categories. Patients with hypertensive emergencies also presented higher blood pressure values than those with hypertensive urgency and hypertensive pseudocrisis. It is also worth noting that there was a significant reduction in the values from the first to the second blood pressure measurement. In the second measurement, only the value of the systolic pressure of patients with hypertensive emergency was higher, when compared to those with hypertensive urgency and hypertensive pseudocrisis. The most frequent clinical manifestations of patients were headache, pain, malaise, chest pain, vertigo, neurological problems, nausea and dyspnea. Less often, cough, emotional problems, such as stress and nervousness, in addition to fever and sweating, were mentioned. In patients with hypertensive emergency compared to those with hypertensive urgency and hypertensive pseudocrisis, neurological problems and dyspnea were more frequent. In patients with hypertensive urgency compared to those with hypertensive emergency and hypertensive pseudocrisis, headache, malaise and chest pain were more frequent. In patients with hypertensive pseudocrisis compared to those with hypertensive urgency and hypertensive emergency, respectively, pain and emotional problems were more frequent. The diagnosis of hypertension was more frequent in patients with hypertensive emergency, compared to those with hypertensive urgency and hypertensive pseudocrisis. Stroke, acute pulmonary edema and acute myocardial infarction were identified only in patients with hypertensive emergency. The diagnosis of *diabetes mellitus* was more frequent in patients with hypertensive urgency than in those with hypertensive emergency.

**Table 1:** Clinical characteristics and signs and symptoms of patients with hypertensive emergency, urgency and pseudocrisis

		Hypertensive emergency	Hypertensive urgency	Hypertensive pseudocrisis	p value
Age, years		62.7±12.9	54.6±14.0	52.0±13.4	<0.001
Systolic/diastolic pressure, mmHg					
	Measurement 1	214.8±29.2/130.8±14.9	197.2±23.6/124.9±9.2	197.7±22.9/124.5±8.1	<0.001/<0.001
	Measurement 2	164.5±31.7/101.5±19.6	156.8±25.2/96.7±14.3	147.6±18.4/93.3±13.5	0.049/0.096
	Measurement 3	140.0±31.6/81.4±8.9	151.9±30.9/93.0±17.9	142.0±13.0/90.0±7.0	0.518/0.229
Sex					0.612
	Male	34 (21.3)	113 (70.6)	13 (8.1)	
	Female	49 (17.8)	199 (72.4)	27 (9.8)	
Symptoms					
	Headache	2 (2.5)	107 (35.9)	6 (15.0)	<0.001
	Pain	2 (2.5)	49 (16.4)	26 (65.0)	<0.001
	Malaise	4 (4.9)	57 (19.1)	3 (7.5)	0.003
	Chest pain	7 (8.6)	53 (17.8)	0 (0)	0.003
	Dizziness	6 (7.4)	47 (15.8)	5 (12.5)	0.149
	Neurologic problems	39 (48.1)	15 (5.0)	0 (0)	<0.001
	Nausea	3 (3.7)	42 (14.1)	7 (17.5)	0.025
	Dyspnea	22 (27.2)	26 (8.7)	2 (5.0)	<0.001
	Emotional problems	2 (2.5)	1 (0.3)	10 (25.0)	<0.001
Medical diagnoses					
	Hypertension	45 (54.2)	105 (33.7)	10 (25.0)	<0.001
	Stroke	33 (40.7)	0 (0)	0 (0)	<0.001
	*Diabetes mellitus*	10 (12.3)	20 (47.6)	1 (16.7)	<0.001
	Acute pulmonary edema	20 (24.7)	0 (0)	0 (0)	<0.001
	Acute myocardial infarction	10 (12.3)	0 (0)	0 (0)	0.040

Results expressed as mean±standard deviation or n (%).

The data presented in table 2 indicate that blood glucose was the most often evaluated test, followed by electrocardiography, more frequent in patients with hypertensive urgency and hypertensive emergency, compared with those with hypertensive pseudocrisis (p<0.05). Computed tomography was more frequent in patients with hypertensive emergency; and electrocardiography was more often used in those with hypertensive urgency and hypertensive emergency compared to those with hypertensive pseudocrisis. Patients with hypertensive emergency were submitted to a higher proportion of laboratory tests, compared to those with hypertensive urgency. Regarding drug treatment, the use of angiotensin converting enzyme inhibitors and calcium channel blockers was predominant in patients with hypertensive urgency; analgesics were predominant in those with hypertensive pseudocrisis; and bronchodilators, insulin, oxygen therapy, sodium nitroprusside and anticonvulsants were predominant in those with hypertensive emergency. The use of anti-inflammatory treatment was similar in patients with pseudocrisis and hypertensive emergency, being less frequent in patients with hypertensive urgency. The use of antiplatelet agents was similar and only occurred in patients with hypertensive urgency or emergency. Regarding the referral after the emergency department, hospital discharge was more frequent in patients with hypertensive pseudocrisis. Hospitalization was more frequent in patients with hypertensive emergency, and the two patients who progressed to death were identified in the hypertensive emergency and urgency groups.

**Table 2:** Medical tests, treatment and referral of patients with com hypertensive emergency, urgency and pseudocrisis

		Hypertensive emergency	Hypertensive urgency	Hypertensive pseudocrisis	p value
Medical tests					
	Electrocardiography	32 (42.1)	69 (46.3)	1 (6.3)	0.009
	Laboratory tests	40 (52.6)	52 (34.9)	7 (43.8)	0.037
	Glycemia	46 (60.5)	70 (47)	8 (50)	0.156
	Cardiac enzymes	21 (27.6)	47 (31.5)	1 (6.3)	0.101
	X-ray	27 (35.5)	37 (24.8)	3 (18.8)	0.168
	Computed tomography	39 (51.3)	13 (8.7)	1 (6.3)	<0.001
Treatment					
	Angiotensin converting enzyme inhibitors	60 (72.3)	254 (81.4)	27 (67.5)	0.043
	Diuretic	47 (56.6)	187 (59.9)	17 (42.5)	0.107
	Analgesic	11 (13.3)	97 (31.1)	21 (52.5)	<0.001
	Anti-inflammatory	25 (30.1)	47 (15.1)	13 (32.5)	0.001
	Benzodiazepine	9 (10.8)	55 (17.6)	8 (20.0)	0.277
	Calcium channel blocker	6 (7.2)	47 (15.1)	2 (5.0)	0.050
	Antiemetic	7 (84)	39 (12.5)	8 (20)	0.189
	Antiplatelet	11 (13.3)	42 (13.5)	0 (0)	0.047
	Bronchodilator	21 (25.3)	26 (8.3)	0 (0)	<0.001
	Coronary vasodilator	9 (10.8)	36 (11.5)	0 (0)	0.077
	Insulin	11 (13.3)	20 (6.4)	1 (2.5)	0.049
	Betablocker	2 (2.4)	13 (4.2)	0 (0)	0.336
	Sodium nitroprusside	7 (8.4)	5 (1.6)	0 (0)	0.002
	Anticonvulsant	8 (9.6)	2 (0.6)	0 (0)	<0.001
Referral					
	Discharge	2 (2.6)	81 (57.0)	13 (59.1)	<0.001
	Admission	65 (85.5)	30 (21.1)	1 (4.5)	<0.001
	Death	1 (1.3)	1 (0.7)	0 (0)	<0.001

Results expressed as n (%).

In the multinomial logistic regression analysis ( Table 3 ), we observed that being older than 60 years was associated with a lower probability of hypertensive pseudocrisis, when compared to patients with hypertensive emergency. The presence of neurological problems was associated with a much smaller probability (1.5.10^-8^) of hypertensive pseudocrisis compared to hypertensive emergency. The presence of pain (except headache and chest pain) increased 55.58 times the patient's probability of presenting hypertensive pseudocrisis compared to patients with hypertensive emergency, and the presence of emotional problems increased 17.13 times the probability of this outcome. When comparing patients who presented hypertensive urgency with those with hypertensive emergency, only the presence of headache increased the probability of this outcome (14.28 times). On the other hand, age over 60 years, and presence of neurological and emotional problems, were protective for the development of hypertensive urgency, compared to hypertensive emergency.

**Table 3:** Multinomial logistic regression of crisis-associated variables hypertensive

Classification of hypertensive crisis		OR	95%CI	p value
Pseudocrisis/emergency				
	Age >60 years	0.32	0.10-0.96	0.042
	Pain	55.58	10.55-292.74	<0.001
	Neurologic problems	1.5.10^-8^	1.5.10^-8^-1.5.10^-8^	<0.001
	Emotional problems	17.13	2.80-104.87	0.002
Urgency/emergency				
	Age >60 years	0.50	0.27-0.92	0.025
	Headache	14.28	3.32-61.47	<0.001
	Neurologic problems	0.09	0.04-0.18	<0.001
	Emotional problems	0.06	4.7.10^-3^-0.79	0.032

OR: odds ratio; 95%CI: 95% confidence interval.

## DISCUSSION

The main finding of the present study was the determining presence of signs and symptoms, such as pain, emotional problems, neurological problems and headache as predictors of hypertensive crisis. The results also showed that six out of 1,000 patients who sought emergency services had hypertensive crises. The classification of the hypertensive crisis in hypertensive emergency, hypertensive urgency or hypertensive pseudocrisis was performed through the information about symptoms contained in the patients' records. Despite the severity of hypertensive emergency due to end-organ damage and risk of death, this condition was not the most frequent. Hypertensive urgency had the highest prevalence in this study, corresponding to 71.7%. These findings are very similar to those found in a multicenter study conducted in ten Italian hospitals, in which the prevalence of hypertensive urgency and hypertensive emergency was 74.7% and 25.3%, respectively.^(^^8^^)^ In the medical practice of emergency care, hypertensive urgency is frequently observed, corroborating once more the data found in the present study. However, when admitted to an emergency department, patients should be treated as if they have a potential risk of death, until clinical and/or laboratory tests are conducted, excluding the possibility of a hypertensive emergency and confirming the hypertensive urgency. An international study with 387 patients presenting hypertensive crisis showed confirmation of hypertensive emergency, after the tests were conducted, in only 10.1% of the patients.^(^^9^^)^

Another category of hypertensive crisis, which is little discussed in the literature and in clinical practice, is hypertensive pseudocrisis. Its definition was described in the Brazilian literature at the Multicenter Meeting on Hypertensive Crises^(^^6^^)^ and in the 7^th^ Brazilian Hypertension Guideline,^(^^10^^)^ that define hypertensive pseudocrisis as a transient elevation of blood pressure, during an emotional, painful or uncomfortable event in patients with complaints of headache, atypical chest pain, dyspnea, acute psychological stress, and panic syndrome. In the present study, the prevalence of this category was less frequent when compared to hypertensive urgencies and hypertensive emergencies. A study carried out in an emergency service addressed three categories of hypertensive crisis, and showed a low prevalence (4%) of hypertensive pseudocrisis, corroborating the findings of the present study.^(^^11^^)^ Since hypertensive pseudocrisis is triggered by painful or emotional events, it is deemed possible to identify it and conduct its treatment based on the symptoms. It is noteworthy that a hypertensive pseudocrisis can be easily confused with a hypertensive urgency, because it does not present end-organ damage, therefore it is essential to have a clear definition of the symptoms.

The profile of the patients studied showed that the mean age was in the fifth decade of life, not differing from other studies that also evaluated the profile of patients with hypertensive crisis.^(^^12^^,^^13^^)^ In the present study, age was a relevant variable because in the multivariate analysis it was associated to hypertensive crisis, reducing by 0.32 times the probability of a hypertensive pseudocrisis, and reducing by 0.50 times the probability of a hypertensive urgency, compared to patients with hypertensive emergency. These results have never been published before, as nothing was found in the literature on the subject with analysis by multinomial logistic regression. Hypertensive crisis is usually classified only as hypertensive urgency and hypertensive emergency. Therefore, multinomial logistic regression allowed the analysis of the three categories of hypertensive crisis simultaneously. Thus, the importance of this study is based on the symptoms presented by the study population, which made possible the hypertensive crisis classification and the identification of its relation with the signs and symptoms expected for each type of the three categories of hypertensive crisis.

Headache was the most frequent clinical manifestation associated with hypertensive crisis, a finding corroborated by studies on patients with hypertensive crisis which also identified headache as the most frequent symptom.^(^^12^^,^^14^^,^^15^^)^ Besides, in the present study, headache presented a higher frequency among patients with hypertensive urgency (35.9%) and remained associated with this category after multinomial analysis, increasing 14.28 times the chance of this symptom when compared to patients with hypertensive emergency. Headache is closely linked to increased blood pressure levels due to a rupture in the brain self-regulating mechanism, which results in vasodilation and increased cerebral blood flow,^(^^16^^)^ which could explain the data found.

Regarding the blood pressure values, the patients studied showed very high baseline levels (200.4±25.3mmHg/125.74±10.5mmHg). However, they were compatible with a much used definition for hypertensive crisis and with the findings of an international study on the clinical manifestations of patients with hypertensive crisis.^(^^17^^)^ This data was expected, since the concept of hypertensive crisis used supports high blood pressure values. However, in the second blood pressure measurement, a reduction in blood pressure levels was observed, presuming the efficacy of the treatment conducted. The present study also showed a reduction in blood pressure levels in patients with hypertensive pseudocrisis. Possibly due to its association with emotional or painful events, it is presumed that there are no potential organic dysfunctions and, once the symptoms were treated, there has been a reduction in blood pressure levels.

In addition, the presence of pain and emotional problems was more frequently associated with hypertensive pseudocrisis, and this association remained after logistic regression analysis, showing that pain (except headache and chest pain) and emotional problems increased the chance of hypertensive pseudocrisis when compared to patients with hypertensive emergency, corroborating the concept of hypertensive pseudocrisis. Besides, the records found in the literature regarding patients with hypertensive pseudocrisis are old and scarce. The analysis of the Brazilian literature revealed two studies published over a decade ago that analyzed the clinical management in situations of hypertensive crisis, including pseudocrisis^(^^18^^)^ and the prevalence and clinical characteristics of patients with hypertensive pseudocrisis.^(^^19^^)^ In Brazil, the lack of studies on clinical characteristics of patients, including hypertensive pseudocrisis, emphasizes the importance of our data. In addition to being unprecedented, our study simultaneously analyzed hypertensive urgency, hypertensive emergency and hypertensive pseudocrisis.

Regarding the clinical manifestations, the most frequent signs and symptoms identified in patients with hypertensive emergency were neurological problems (48.1%) and dyspnea (27.2%), suggesting the presence of end-organ damage, which is characteristic of hypertensive emergency. A study on the subject has shown that among the major findings are pathophysiological changes occuring in the body in case of high blood pressure, such as the self-regulation to maintain adequate and stable blood flow to the brain, heart and kidneys during pressure fluctuations.^(^^20^^)^ In addition, the inflammatory process is part of the pathophysiology of arterial hypertension, triggering complex interactions in the organic systems, such as the central nervous system and the cardiovascular system. This reinforces the concept of hypertensive emergency whereby the signs and symptoms evidence end-organ damage. In the present study, we also observed a close relation between medical diagnoses of stroke, acute lung edema and myocardial infarction in patients with hypertensive emergency, suggesting the presence of end-organ damage.

Relevant and clearly described in the literature is the importance of identification of symptoms for differentiating the type of hypertensive crisis, since this will affect the assertive treatment. Specific tests for end-organ damage showed a significant association, as expected, in laboratory tests (p=0.037), electrocardiography (p=0.00); and computed tomography (p<0.001). A study about the subject points out the main finding of fast differentiation between hypertensive urgency and hypertensive emergency, so that the choice of treatment depends on the clinical presentation of the patient.^(^^21^^)^ Also, laboratory tests, electrocardiogram, chest x-ray, among other examinations, are part of the patient's evaluation for the diagnosis of stroke, and an imaging test is indicated.^(^^22^^)^ In the emergency department of the present study, a computed tomography was performed whenever a stroke was suspected.

Considering treatment of the hypertensive crisis, the findings of the use of hypotensive, analgesic, anti-inflammatory and anticonvulsant drugs, are consistent with the literature,^(^^23^^,^^24^^)^ since their main objective is to reduce pressure levels as initial treatment.^(^^3^^)^ As to patient's referral after the emergency room care, as expected, patients with hypertensive pseudocrisis and hypertensive urgency were discharged more frequently. Possibly for not presenting end-organ damage, these patients received adequate treatment and were discharged. On the other hand, patients with hypertensive emergency, due to their imminent risk of life and end-organ damage, required treatment and hospitalization.

As a limitation of the study, because this was an analysis of the clinical characteristics associated to the hypertensive crisis through the patients' emergency department records, the data collection showed weaknesses in the structure of the emergency service where the study was performed. Due to the lack of a computerized system, the information in the emergency department records was entered manually, and sometimes it was difficult to read, providing few independent variables to the study, and this fact may also have implied underestimated prevalences. However, the recognition of the factors associated with hypertensive crisis, as well as the clinical profile of patients, can provide subsidies for changes in the screening and referral processes of patients with hypertensive crisis in healthcare services.

## CONCLUSION

Symptoms presented by patients in emergency services are considered paramount for the outcome of the hypertensive crisis, and they can prevent the severe progression of this hypertensive complication. Our results deserve consideration and may contribute to the improvement of clinical practice, mainly due to the possibility of classifying the hypertensive crisis as hypertensive emergency, hypertensive urgency or hypertensive pseudocrisis, in emergency services.
