# Supplementary material for: Hypertensive crisis: clinical characteristics of patients with hypertensive urgency, emergency and pseudocrisis at a public emergency department
Source: Einstein (Sao Paulo). 2019 Aug 27;17(4):eAO4685. doi: 10.31744/einstein_journal/2019AO4685 (PMC6711750; doi:10.31744/einstein_journal/2019AO4685)
Supplement: Translation

# Translation: Crise hipertensiva: características clínicas de pacientes com urgência, emergência e pseudocrise hipertensivas em um serviço público de emergência

**Authors:** Angela Maria Geraldo Pierin, Carime Farah Flórido, Juliano dos Santos

**Affiliations:** 1 Universidade de São Paulo, São Paulo, SP, Brasil.; 2 Escola de Enfermagem, Universidade de São Paulo, São Paulo, SP, Brasil.; 3 Instituto Nacional de Câncer, Rio de Janeiro, RJ, Brasil.

**Keywords:** Hipertensão, Serviços médicos de emergência, Tratamento de emergência, Prevalência

## Abstract

**Objetivo:**

Avaliar pacientes com crise hipertensiva, classificada em urgência, emergência ou pseudocrise, e identificar variáveis associadas.

**Métodos:**

Foram avaliados 508 pacientes (57% mulheres; 56,3±13,8 anos) com crise hipertensiva (pressão diastólica de 120mmHg), idade maior ou igual a 18 anos, atendidos em um serviço de emergência de um hospital geral público. A crise hipertensiva foi classificada em urgência, emergência ou pseudocrise.

**Resultados:**

A prevalência da crise hipertensiva foi 6/1.000, com 71,7% com urgência hipertensiva, 19,1% com emergência hipertensiva e 9,2% com pseudocrise hipertensiva. Na análise de regressão logística multinomial, as condições de pseudocrise e urgência foram comparadas com a emergência hipertensiva. Assim, presença de dor (OR: 55,58; IC95%: 10,55-292,74), exceto precordialgia e cefaleia, e problemas emocionais (OR: 17,13; IC95%: 2,80-104,87) elevaram a chance para pseudocrise hipertensiva. Idade acima de 60 anos (OR: 0,32; IC95%: 0,10-0,96) e problemas neurológicos (OR: 1,5.10^-8^; IC95%: 1,5.10^-8^-1,5.10^-8^) foram protetores para pseudocrise hipertensiva. A urgência hipertensiva comparada com emergência hipertensiva mostrou que idade acima de 60 anos (OR: 0,50; IC95%: 0,27-0,92), problemas neurológicos (OR: 0,09; IC95%: 0,04-0,18) e emocionais (OR: 0,06; IC95%: 4,7.10^-3^-0,79) foram protetores para urgência hipertensiva, e apenas cefaleia (OR: 14,28; IC95%: 3,32-61,47) elevou a chance para urgência hipertensiva.

**Conclusão:**

Idade mais elevada e problemas neurológicos se associaram à emergência hipertensiva. Cefaleia associou-se à urgência hipertensiva. Dor e problemas emocionais se associaram à pseudocrise hipertensiva. Nossos resultados podem contribuir para aprimorar a identificação de pacientes com crise hipertensiva que procuram serviços de emergência.

## INTRODUÇÃO

A Organização Mundial da Saúde aponta que as doenças cardiovasculares são responsáveis por aproximadamente 17 milhões de morte por ano, e as complicações decorrentes da hipertensão arterial respondem por 9,4 milhões dessas mortes.^(^^1^^)^A crise hipertensiva está entre as principais complicações decorrentes da hipertensão arterial que levam à procura pelos serviços de emergência.^(^^2^^)^Em artigo de revisão sobre o assunto, demonstra-se que a prevalência e as características dos pacientes com crise hipertensiva têm se modificado nas últimas quatro décadas. Porém, a morbidade e a mortalidade ainda são significativas.^(^^3^^)^

A crise hipertensiva é caracterizada por severa e abrupta elevação da pressão arterial,^(^^4^^)^geralmente definida por valores da pressão diastólica acima de 120mmHg. É classificada em urgência hipertensiva quando não há lesões de órgãos-alvo e em emergência hipertensiva quando há risco de vida evidenciado por lesão de órgãos-alvo.^(^^5^^)^A urgência e a emergência hipertensiva devem ser diferenciadas da pseudocrise hipertensiva, que é caracterizada pela elevação transitória da pressão arterial diante de eventos dolorosos ou emocionais, como cefaleia, tontura rotatória, ansiedade ou síndrome do pânico.^(^^6^^)^O tratamento da urgência hipertensiva consiste em redução gradativa da pressão arterial com medicamento oral, enquanto na emergência hipertensiva é indicado medicamento intravenoso para redução mais rápida da pressão arterial.^(^^7^^)^A pseudocrise tem seu tratamento com o foco na sintomatologia e é pouco explorada na literatura que aborda a crise hipertensiva.

A literatura brasileira ainda é pobre quanto a estudos relacionados às características das pessoas com crise hipertensiva nos serviços de emergências e seus fatores associados. A atuação da equipe multiprofissional envolve articulações para o manejo correto no atendimento devido à complexidade das situações de urgência e emergência apresentada por essa condição clínica, e a falta de conhecimento da crise hipertensiva pode causar impacto inesperado na equipe de saúde. Desta forma, considera-se que o presente estudo seja relevante por promover a elucidação do tema, fortalecendo o conhecimento nacional.

## OBJETIVO

Avaliar pacientes com crise hipertensiva, classificada em urgência, emergência ou pseudocrise hipertensiva; e identificar as variáveis associadas.

## MÉTODOS

Realizou-se estudo transversal, retrospectivo, com abordagem quantitativa, em um serviço de emergência de um hospital municipal da cidade de São Vicente, localizada na costa sul do litoral do Estado de São Paulo. A cidade de São Vicente possui população fixa em torno de 350 mil habitantes. O hospital é público, possui unidade de emergência especializada em atendimento de urgência e emergência adulto e pediátrico. Para identificar os pacientes com crise hipertensiva foram avaliadas todas as fichas dos pacientes atendidos no serviço de emergência para adulto e na especialidade clínica, no período janeiro a junho de 2015, no total de 83.774 atendimentos.

Foram identificados 508 pacientes que atenderam os seguintes critérios de inclusão: pressão arterial diastólica ≥120mmHg e idade ≥18 anos. Dos 508 pacientes identificados, 435 foram incluídos e 73 excluídos devido à ausência de registros nas fichas de atendimento, que não possibilitou identificar as variáveis do estudo e nem a classificação do tipo de crise hipertensiva.

As variáveis independentes estudadas foram idade, sexo, valores da pressão arterial, motivo da procura ao serviço, tratamento, diagnóstico médico identificado e destino. As variáveis desfecho foram emergência hipertensiva, urgência hipertensiva e pseudocrise hipertensiva. A classificação da crise hipertensiva foi realizada por especialistas, de acordo com o *Seventh report of the Joint National Committee on Prevention, Detection, Evaluation, and Treatment of High Blood Pressure* (JNC 7),^(^^5^^)^com atenção e senso crítico baseando-se principalmente nos sinais e sintomas dos pacientes. Foram considerados com pseudocrise hipertensiva pacientes que apresentaram os sinais e sintomas como dor de origem musculoesquelética, gastralgias, odenofagias, pirose e dores de modo geral, exceto precordialgia e cefaleia não especificada, as quais entraram na classificação de urgência ou emergência hipertensiva, a depender de outros sinais e sintomas, que evidenciassem ou não lesões de órgãos-alvo, e/ou tinham registro de diagnóstico médico que permitisse a identificação de problemas emocionais, como picos hipertensivos pós-estresse e labilidade emocional. Foram classificados como emergência hipertensiva, pacientes que apresentaram sinais e sintomas evidenciando lesões de órgãos-alvo como problemas neurológicos, incluindo diminuição da força motora, parestesia, confusão, desorientação mental, precordialgia e dispneia, além dos diagnósticos médicos acidente vascular encefálico, infarto agudo do miocárdio e edema agudo de pulmão. As urgências hipertensivas foram classificadas de acordo com sinais e sintomas que não caracterizaram pseudocrise hipertensiva ou não apresentaram lesões de órgãos-alvo. A coleta de dados foi realizada por meio dos registros das fichas de atendimento. Desta forma, após a realização dos exames laboratoriais, como rotina do serviço de emergência, os pacientes foram reavaliados pela equipe médica, que procedeu realizando o registro do diagnóstico corresponde às alterações dos exames, permitindo a classificação da crise hipertensiva em urgência ou emergência no presente estudo.

Para caracterizar a pressão arterial, foram considerados os valores registrados na ficha do serviço de emergência. A variável foi expressa pela média e desvio padrão em mmHg. A medida da pressão arterial, no serviço de emergência no qual foi realizado o estudo, rotineiramente é feita por médicos e pela equipe de enfermagem, pelo método indireto, com técnica auscultatória e uso de aparelhos aneroides, cuja calibração é avaliada periodicamente. Para a identificação das manifestações clínicas, das condutas e do tratamento dos pacientes, foram considerados todos os registros da ficha de atendimento de emergência, por leitura cuidadosa e criteriosa, por duas enfermeiras que atuavam no serviço. O estudo foi aprovado pelo Comitê de Ética, CAAE: 53896016.9.0000.5392, além de ter havido concordância da chefia do serviço de emergência.

Os resultados foram expressos como média±desvio padrão (para variáveis de distribuição normal), e números absolutos e percentuais para as variáveis categóricas. Para os dados paramétricos, usou-se análise de variância (ANOVA). Utilizou-se o coeficiente de Pearson para avaliação de proporções. Valores de p≤0,05 foram considerados estatisticamente significativos. Realizou-se análise multivariada por regressão logística multinomial para identificar as variáveis independentes que se mantiveram associadas à crise hipertensiva, como idade acima de 60 anos, dor (exceto cefaleia e precordialgia), cefaleia, problemas neurológicos e problemas emocionais, sobre as chances de desenvolver pseudocrise ou urgência hipertensiva em relação à emergência hipertensiva. Nesse modelo, o teste estatístico utilizado foi o χ^2^de Pearson. Os dados obtidos foram inseridos em planilha do Microsoft Excel^®^utilizando o *Statistical Package for Social Science* (SPSS), versão 22, e o programa R versão 3.3.3.

## RESULTADOS

Do total de pacientes avaliados retrospectivamente no serviço de emergência, no período de 6 meses, a prevalência da crise hipertensiva foi 6/1.000. A maioria foi classificada como urgência hipertensiva, seguida de emergência e pseudocrise hipertensiva, conforme mostrado na figura 1 .

**Figura 1:** População incluída no estudo

Os dados da tabela 1 mostram que a maioria dos pacientes era do sexo feminino, e a média de idade encontrada esteve na faixa da quinta década. Os pacientes com emergência hipertensiva apresentaram idade significativamente mais elevada do que aqueles com urgência hipertensiva e pseudocrise hipertensiva, porém não houve diferença significativa em relação ao sexo nessas três categorias. Os pacientes com emergência hipertensiva também apresentaram valores da pressão arterial da primeira medida mais elevados do que os com urgência e pseudocrise hipertensiva. Destaca-se, ainda, que houve redução significativa nos valores da primeira para a segunda medida da pressão arterial. Na segunda medida, apenas o valor da pressão sistólica dos pacientes com emergência hipertensiva foi mais elevado, quando comparado àqueles com urgência e pseudocrise hipertensiva. As manifestações clínicas dos pacientes mais frequentes foram cefaleia, dor, mal-estar, precordialgia, vertigem, problemas neurológicos, náuseas e dispneia. Em frequências menores, foram citados tosse, problemas emocionais como estresse e nervosismo, além de febre e sudorese. Nos pacientes com emergência hipertensiva comparados aos com urgência e pseudocrise hipertensiva foram mais frequentes os problemas neurológicos e a dispneia. Nos pacientes com urgência hipertensiva comparados aos com emergência e pseudocrise hipertensiva, foram mais frequentes cefaleia, mal-estar e dor torácica. Nos pacientes com pseudocrise hipertensiva comparados aos com emergência e urgência hipertensiva, respectivamente, foram mais frequentes dor e problemas emocionais. O registro do diagnóstico de hipertensão arterial foi mais frequente nos pacientes com emergência hipertensiva, comparados aos com urgência e pseudocrise hipertensiva. Acidente vascular encefálico, edema agudo de pulmão e infarto agudo do miocárdio foram identificados apenas nos pacientes com emergência hipertensiva. O diagnóstico de *diabetes mellitus* foi mais frequente nos pacientes com urgência hipertensiva em relação aos com emergência hipertensiva.

**Tabela 1:** Características clínicas e sinais e sintomas dos pacientes com emergência, urgência e pseudocrise hipertensiva

		Emergência hipertensiva	Urgência hipertensiva	Pseudocrise hipertensiva	Valor de p
Idade, anos		62,7±12,9	54,6±14,0	52,0±13,4	<0,001
Pressão sistólica/diastólica, mmHg					
	Medida 1	214,8±29,2/130,8±14,9	197,2±23,6/124,9±9,2	197,7±22,9/124,5±8,1	<0,001/<0,001
	Medida 2	164,5±31,7/101,5±19,6	156,8±25,2/96,7±14,3	147,6±18,4/93,3±13,5	0,049/0,096
	Medida 3	140,0±31,6/81,4± 8,9	151,9±30,9/93,0±17,9	142,0±13,0/90,0±7,0	0,518/0,229
Sexo					0,612
	Masculino	34 (21,3)	113 (70,6)	13 (8,1)	
	Feminino	49 (17,8)	199 (72,4)	27 (9,8)	
Sintomas					
	Cefaleia	2 (2,5)	107 (35,9)	6 (15,0)	<0,001
	Dor	2 (2,5)	49 (16,4)	26 (65,0)	<0,001
	Mal-estar	4 (4,9)	57 (19,1)	3 (7,5)	0,003
	Dor torácica	7 (8,6)	53 (17,8)	0 (0)	0,003
	Tontura	6 (7,4)	47 (15,8)	5 (12,5)	0,149
	Problemas neurológicos	39 (48,1)	15 (5,0)	0 (0)	<0,001
	Náusea	3 (3,7)	42 (14,1)	7 (17,5)	0,025
	Dispneia	22 (27,2)	26 (8,7)	2 (5,0)	<0,001
	Problemas emocionais	2 (2,5)	1 (0,3)	10 (25,0)	<0,001
Diagnósticos médicos					
	Hipertensão arterial	45 (54,2)	105 (33,7)	10 (25,0)	<0,001
	Acidente vascular encefálico	33 (40,7)	0 (0)	0 (0)	<0,001
	*Diabetes mellitus*	10 (12,3)	20 (47,6)	1 (16,7)	<0,001
	Edema agudo de pulmão	20 (24,7)	0 (0)	0 (0)	<0,001
	Infarto agudo do miocárdio	10 (12,3)	0 (0)	0 (0)	0,040

Resultados expressos por média±desvio padrão ou n (%).

Os dados apresentados na tabela 2 indicam que a glicemia capilar foi a avaliação mais realizada, seguida de eletrocardiograma, mais presente nos pacientes com urgência e emergência, comparados aos com pseudocrise hipertensiva (p<0,05). A realização de tomografia computadorizada foi mais frequente nos pacientes com emergência hipertensiva e eletrocardiograma naqueles em situação de urgência e emergência hipertensiva comparados aos com pseudocrise hipertensiva. Os pacientes com emergência hipertensiva realizaram em maior proporção exames laboratoriais, comparados àqueles com urgência hipertensiva. Em relação ao tratamento medicamentoso, verificou-se o predomínio do uso de inibidores da enzima conversora de angiotensina e os bloqueadores dos canais de cálcio nos pacientes com urgência hipertensiva; analgésicos naqueles com pseudocrise hipertensiva; e broncodilatadores, insulina, oxigenoterapia, nitroprussiato de sódio e anticonvulsivante na situação de emergência hipertensiva. O tratamento com anti-inflamatórios foi similar nos pacientes com pseudocrise e emergência hipertensiva; e teve menor frequência na urgência hipertensiva. O uso de antiagregante plaquetário foi similar e apenas ocorreu nos pacientes com urgência ou emergência hipertensiva. Quanto ao destino após o atendimento no serviço de emergência, a alta foi mais frequente nos pacientes com pseudocrise hipertensiva. A internação esteve mais presente nos pacientes com emergência hipertensiva, e os dois pacientes que evoluíram para óbito foram identificados nos grupos emergência e urgência hipertensiva.

**Tabela 2:** Exames médicos, tratamento e encaminhamento dos pacientes com emergência, urgência e pseudocrise hipertensiva

		Emergência hipertensiva	Urgência hipertensiva	Pseudocrise hipertensiva	Valor de p
Exames médicos					
	Eletrocardiograma	32 (42,1)	69 (46,3)	1 (6,3)	0,009
	Exames laboratoriais	40 (52,6)	52 (34,9)	7 (43,8)	0,037
	Glicemia	46 (60,5)	70 (47)	8 (50)	0,156
	Enzimas cardíacas	21 (27,6)	47 (31,5)	1 (6,3)	0,101
	Raio X	27 (35,5)	37 (24,8)	3 (18,8)	0,168
	Tomografia computadorizada	39 (51,3)	13 (8,7)	1 (6,3)	<0,001
Tratamento					
	Inibidores da enzima conversora de angiotensina	60 (72,3)	254 (81,4)	27 (67,5)	0,043
	Diurético	47 (56,6)	187 (59,9)	17 (42,5)	0,107
	Analgésico	11 (13,3)	97 (31,1)	21 (52,5)	<0,001
	Anti-inflamatório	25 (30,1)	47 (15,1)	13 (32,5)	0,001
	Benzodiazepínico	9 (10,8)	55 (17,6)	8 (20,0)	0,277
	Bloqueador dos canais de cálcio	6 (7,2)	47 (15,1)	2 (5,0)	0,050
	Antiemético	7 (84)	39 (12,5)	8 (20)	0,189
	Antiagregante plaquetário	11 (13,3)	42 (13,5)	0 (0)	0,047
	Broncodilatador	21 (25,3)	26 (8,3)	0 (0)	<0,001
	Vasodilatador coronariano	9 (10,8)	36 (11,5)	0 (0)	0,077
	Insulina	11 (13,3)	20 (6,4)	1 (2,5)	0,049
	Betabloqueador	2 (2,4)	13 (4,2)	0 (0)	0,336
	Nitroprussiato de sódio	7 (8,4)	5 (1,6)	0 (0)	0,002
	Anticonvulsivante	8 (9,6)	2 (0,6)	0 (0)	<0,001
Encaminhamento					
	Alta	2 (2,6)	81 (57,0)	13 (59,1)	<0,001
	Internação	65 (85,5)	30 (21,1)	1 (4,5)	<0,001
	Óbito	1 (1,3)	1 (0,7)	0 (0)	<0,001

Resultados expressos por n (%).

Na análise de regressão logística multinomial ( Tabela 3 ), verificou-se que o fato de apresentar idade acima de 60 anos se associou à menor chance para desenvolver pseudocrise hipertensiva, comparado aos pacientes que apresentaram emergência hipertensiva. Presença de problemas neurológicos manteve-se associada a chances bem menores (1,5.10^-8^) para ocorrência da pseudocrise hipertensiva em relação à emergência hipertensiva. A presença de dor (exceto cefaleia e precordialgia) aumentou em 55,58 vezes a chance de o paciente apresentar pseudocrise hipertensiva comparados aos pacientes com emergência hipertensiva, além de os problemas emocionais aumentarem em 17,13 vezes a chance desse desfecho. Na comparação dos pacientes que apresentaram urgência hipertensiva em relação àqueles com emergência hipertensiva, apenas a presença de cefaleia aumentou a chance para tal (14,28 vezes). Por outro lado, idade acima de 60 anos, presença de problemas neurológicos e de problemas emocionais, mostraram-se protetores para o desenvolvimento da urgência hipertensiva, comparada à emergência hipertensiva.

**Tabela 3:** Regressão logística multinomial de variáveis associadas à crise hipertensiva

Classificação da crise hipertensiva		OR	IC95%	Valor p
Pseudocrise/emergência				
	Idade >60 anos	0,32	0,10-0,96	0,042
	Dor	55,58	10,55-292,74	<0,001
	Problemas neurológicos	1,5.10^-8^	1,5.10^-8^-1,5.10^-8^	<0,001
	Problemas emocionais	17,13	2,80-104,87	0,002
Urgência/emergência				
	Idade >60 anos	0,50	0,27-0,92	0,025
	Cefaleia	14,28	3,32-61,47	<0,001
	Problemas neurológicos	0,09	0,04-0,18	<0,001
	Problemas emocionais	0,06	4,7.10^-3^-0,79	0,032

OR: *odds ratio* ; IC95%: intervalo de confiança de 95%.

## DISCUSSÃO

O principal achado do presente estudo foi a presença determinante dos sinais e sintomas como dor, problemas emocionais, problemas neurológicos e cefaleia como preditores para a crise hipertensiva. Os resultados mostraram também que seis em cada mil pacientes que procuraram o serviço de emergência apresentaram crise hipertensiva. A classificação da crise hipertensiva em emergência, urgência ou pseudocrise hipertensiva foi realizada por meio da sintomatologia registrada nas fichas de atendimento. Apesar da gravidade da emergência hipertensiva devido a lesões de órgãos-alvo e risco de vida, essa condição não foi a mais frequente. A urgência hipertensiva foi a categoria de maior prevalência encontrada neste estudo, correspondendo a 71,7%. Esses achados são bem próximos aos de estudo multicêntrico realizado em dez hospitais italianos, no qual as prevalências da urgência e da emergência hipertensiva foram 74,7% e 25,3%, respectivamente.^(^^8^^)^Na prática clínica, nos serviços de emergência, urgência hipertensiva é frequentemente observada, justificando mais uma vez os dados encontrados no presente estudo. Contudo, quando admitidos no serviço de emergência, os pacientes devem ser atendidos como se apresentassem potencial risco de vida, até a realização de exames clínicos e/ou laboratoriais, em que seja excluída a possibilidade de emergência hipertensiva e confirmada a urgência hipertensiva. Estudo internacional com 387 pacientes na situação de crise hipertensiva, após a realização de exames, mostrou a confirmação da emergência hipertensiva em apenas 10,1%.^(^^9^^)^

Outra categoria da crise hipertensiva, porém pouco abordada na literatura e na prática clínica, é a pseudocrise hipertensiva. Sua definição foi descrita na literatura brasileira no Encontro Multicêntrico sobre Crises Hipertensivas^(^^6^^)^e na 7ª Diretriz Brasileira de Hipertensão Arterial,^(^^10^^)^que definem pseudocrise hipertensiva como elevação transitória da pressão arterial, diante de evento emocional, doloroso ou desconfortável, em pacientes com queixas de cefaleia, dor torácica atípica, dispneia, estresse psicológico agudo e síndrome de pânico. No presente estudo, a prevalência desta categoria foi a menos frequente, quando comparada às urgências e às emergências hipertensivas. Estudo realizado em serviço de emergência que abordou as três categorias da crise hipertensiva também mostrou baixa prevalência (4%) da pseudocrise hipertensiva, corroborando achados do presente estudo.^(^^11^^)^Considera-se que, na pseudocrise hipertensiva, por ser desencadeada por eventos dolorosos ou emocionais, é possível identificá-la e conduzir o tratamento baseado na sintomatologia. Um fato a ser destacado é que a pseudocrise hipertensiva pode ser facilmente confundida com a urgência hipertensiva, por não apresentar lesões de órgãos-alvo, tornando imprescindível a clara definição da sintomatologia.

O perfil dos pacientes estudados mostrou que a média de idade se manteve na faixa da quinta década, não diferindo de achados de outros estudos que também avaliaram o perfil de pessoas com crise hipertensiva.^(^^12^^,^^13^^)^No presente estudo, a idade foi variável relevante, pois, na análise multivariada, ela manteve-se associada à crise hipertensiva, reduzindo a chance em 0,32 vez para o desenvolvimento de pseudocrise hipertensiva e em 0,50 vez a chance de urgência hipertensiva, em relação aos pacientes com emergência hipertensiva. Estes resultados são inéditos, tendo em vista a não identificação de achados na literatura sobre o assunto com análise por regressão logística multinomial. Ressalta-se que a crise hipertensiva é categorizada usualmente somente em urgência e emergência hipertensiva. Dessa forma, a regressão logística multinomial permitiu a análise das três categorias da crise hipertensiva de forma simultânea. Assim, a importância deste estudo baseia-se na sintomatologia apresentada pela população estudada, o que tornou possível a classificação da crise hipertensiva e sua relação entre os sinais e sintomas esperados para cada tipo das três categorias da crise hipertensiva.

A cefaleia foi a manifestação clínica mais frequente associada à crise hipertensiva, dado corroborado por estudos que caracterizaram os pacientes estudados com crise hipertensiva e também identificaram a cefaleia como sintoma mais frequente.^(^^12^^,^^14^^,^^15^^)^Ainda, no presente estudo, a cefaleia apresentou maior frequência entre os pacientes com urgência hipertensiva (35,9%) e manteve-se associada a essa categoria após análise multinomial, aumentando em 14,28 vezes a chance desse sintoma em relação aos pacientes com emergência hipertensiva. A cefaleia está intimamente ligada ao aumento dos níveis pressóricos, devido à ruptura no mecanismo autorregulador cerebral, que resulta em vasodilatação e aumento do fluxo sanguíneo cerebral,^(^^16^^)^o que poderia explicar o dado encontrado.

Em relação aos valores pressóricos, os pacientes estudados mostraram níveis iniciais bastante elevados (200,4±25,3mmHg/125,74±10,5mmHg), porém compatíveis com a definição muito usada para crise hipertensiva e de achados de estudo internacional, que abordou as manifestações clínicas de pacientes com crise hipertensiva.^(^^17^^)^Este dado era esperado, pois o conceito de crise hipertensiva utilizado sustenta valores elevados de pressão arterial. Contudo, na segunda medida da pressão, observou-se redução nos níveis pressóricos, supondo a eficácia do tratamento realizado. O presente estudo mostrou também redução nos níveis pressóricos nos pacientes com pseudocrise hipertensiva. Possivelmente por estar associada a eventos emocionais ou dolorosos, supõe-se que não ocorram disfunções orgânicas em potencial e, uma vez tratada a sintomatologia, houve redução nos níveis pressóricos.

Além disso, a presença de dor e de problemas emocionais esteve associada em maior frequência à pseudocrise hipertensiva, mantendo-se associada após análise de regressão logística, mostrando que dor (exceto cefaleia e precordialgia) e problemas emocionais aumentaram a chance do desenvolvimento de pseudocrise hipertensiva em relação aos pacientes com emergência hipertensiva e corroborando o conceito de pseudocrise hipertensiva. Ainda, os registros encontrados na literatura a respeito de pacientes com pseudocrise hipertensiva são antigos e escassos. A análise da literatura nacional revelou dois estudos publicados há cerca de uma década, que analisaram a conduta médica em situações de crise hipertensiva, incluindo a pseudocrise^(^^18^^)^e a prevalência e características clínicas de pacientes com pseudocrise hipertensiva.^(^^19^^)^Portanto, em função da escassez de estudos, em nosso meio, dentre as análises de características clínicas de pacientes, incluindo a pseudocrise hipertensiva, ressalta-se a importância dos nossos dados que, além de inéditos, analisaram simultaneamente a urgência, a emergência e a pseudocrise hipertensiva.

Em relação às manifestações clínicas, os sinais e sintomas mais frequentes identificados nos pacientes com emergência hipertensiva foram problemas neurológicos (48,1%) e dispneia (27,2%), sugerindo a presença de lesões de órgãos-alvo, característica da emergência hipertensiva. Estudo sobre o assunto mostrou que estão entre os principais achados as mudanças fisiopatológicas que ocorrem no organismo diante da pressão arterial elevada, como a autorregulação que se destina a manter um fluxo sanguíneo adequado e estável para o cérebro, coração e rins durante as flutuações da pressão arterial.^(^^20^^)^Ademais, o processo inflamatório faz parte da fisiopatologia da hipertensão arterial, desencadeando interações complexas nos sistemas orgânicos, como sistema nervoso central e cardiovascular. Reforça-se, então, o conceito sobre emergências hipertensivas segundo o qual os sinais e sintomas evidenciam lesões de órgãos-alvo. Nota-se, ainda, no presente estudo, estreita relação entre os diagnósticos médicos acidente vascular encefálico, edema agudo de pulmão e infarto do miocárdio nos pacientes com emergência hipertensiva, sugerindo a presença de lesões de órgãos-alvo.

Aspecto relevante e claramente descrito na literatura é a importância da identificação da sintomatologia para a diferenciação do tipo de crise hipertensiva, pois cursará no tratamento assertivo. Os exames específicos para lesões de órgãos-alvo apresentaram associação significativa, como esperado, na realização de exames laboratoriais (p=0,037), eletrocardiograma (p=0,009) e tomografia computadorizada (p<0,001). Estudo sobre o assunto aponta como principal achado a rápida identificação da diferenciação da urgência e da emergência hipertensiva, de modo que a escolha do tratamento depende da apresentação clínica do paciente.^(^^21^^)^Ainda, análises laboratoriais, realização de eletrocardiograma, raio X de tórax, entre outros exames, fazem parte da avaliação do paciente, para o diagnóstico de acidente vascular encefálico, sendo indicada a realização de exame de imagem.^(^^22^^)^No serviço de emergência do presente estudo, a realização de tomografia computadorizada ocorreu na suspeita de acidente vascular encefálico.

Em relação ao tratamento da crise hipertensiva, os achados do uso de medicamentos hipotensores, analgésicos, anti-inflamatórios e anticonvulsivantes, condizem com a literatura,^(^^23^^,^^24^^)^pois têm como principal objetivo a redução nos níveis pressóricos como tratamento inicial.^(^^3^^)^Quanto ao destino do paciente após o atendimento no serviço de emergência, como esperado, os pacientes com pseudocrise e urgência hipertensiva receberam alta em maior frequência. Possivelmente por não apresentarem lesões de órgãos-alvo, receberam tratamento adequado e alta hospitalar. Em contrapartida, pacientes com emergência hipertensiva, por apresentarem risco iminente de vida e lesões de órgãos-alvo, necessitaram tratamento e internação hospitalar.

Como limitação do estudo, por se tratar de análise de características clínicas associadas à crise hipertensiva por meio de registros de fichas de atendimento, a coleta de dados mostrou fragilidades na estrutura do serviço de emergência onde o estudo foi realizado. Por não dispor de sistema informatizado, os registros nas fichas de atendimento eram realizados de forma manual, o que dificultou a leitura de alguns dados, fornecendo poucas variáveis independentes ao estudo, e esse fato também pode ter implicado em prevalências subestimadas. Contudo, o reconhecimento dos fatores associados à crise hipertensiva, bem como o perfil clínico dos pacientes, pode fornecer subsídios para mudanças nos processos de triagem e encaminhamento dos pacientes com crise hipertensiva nos serviços de saúde.

## CONCLUSÃO

Considera-se que a sintomatologia apresentada pelos pacientes em serviços de emergência é de fundamental importância para o desfecho da crise hipertensiva, podendo prevenir o grave curso dessa complicação hipertensiva. Nossos resultados merecem consideração e podem contribuir para o aprimoramento da prática clínica, principalmente pela possibilidade da categorização da crise hipertensiva em emergência, urgência e pseudocrise, em serviços de emergência.
